# Deep learning to detect acute respiratory distress syndrome on chest radiographs: a retrospective study with external validation

**DOI:** 10.1016/S2589-7500(21)00056-X

**Published:** 2021-04-20

**Authors:** Michael W Sjoding, Daniel Taylor, Jonathan Motyka, Elizabeth Lee, Ivan Co, Dru Claar, Jakob I McSparron, Sardar Ansari, Meeta Prasad Kerlin, John P Reilly, Michael G S Shashaty, Brian J Anderson, Tiffanie K Jones, Harrison M Drebin, Caroline A G Ittner, Nuala J Meyer, Theodore J Iwashyna, Kevin R Ward, Christopher E Gillies

**Affiliations:** Division of Pulmonary and Critical Care Medicine, Department of Internal Medicine (M W Sjoding MD, I Co MD, D Claar MD, J I McSparron MD, Prof T J Iwashyna MD), Center for Computational Medicine and Bioinformatics (M W Sjoding, Prof K R Ward MD), Department of Radiology (E Lee MD), and Department of Emergency Medicine (I Co, S Ansari PhD, Prof K R Ward, C E Gillies PhD), University of Michigan Medical School, Ann Arbor, MI, USA; Michigan Center for Integrative Research in Critical Care; Ann Arbor, MI, USA (M W Sjoding, D Taylor MS, J Motyka MS, I Co, J I McSparron, S Ansari, Prof K R Ward, C E Gillies); Institute for Healthcare Policy and Innovation (M W Sjoding), and Michigan Institute for Data Science (C E Gillies), University of Michigan, Ann Arbor, MI, USA; Division of Pulmonary, Allergy, and Critical Care Medicine, Department of Medicine (M P Kerlin MD, J P Reilly MD, M G S Shashaty MD, B J Anderson MD, T K Jones MD, H M Drebin MD, C A G Ittner MD, N J Meyer MD), and Center for Translational Lung Biology (J P Reilly, T K Jones, H M Drebin, C A G Ittner, N J Meyer), University of Pennsylvania Perelman School of Medicine, Philadelphia, PA, USA; VA Center for Clinic Management Research, Ann Arbor, MI, USA (Prof T J Iwashyna); Institute for Social Research, Ann Arbor, MI, USA (Prof T J Iwashyna)

## Abstract

**Background:**

Acute respiratory distress syndrome (ARDS) is a common, but under-recognised, critical illness syndrome associated with high mortality. An important factor in its under-recognition is the variability in chest radiograph interpretation for ARDS. We sought to train a deep convolutional neural network (CNN) to detect ARDS findings on chest radiographs.

**Methods:**

CNNs were pretrained on 595 506 radiographs from two centres to identify common chest findings (eg, opacity and effusion), and then trained on 8072 radiographs annotated for ARDS by multiple physicians using various transfer learning approaches. The best performing CNN was tested on chest radiographs in an internal and external cohort, including a subset reviewed by six physicians, including a chest radiologist and physicians trained in intensive care medicine. Chest radiograph data were acquired from four US hospitals.

**Findings:**

In an internal test set of 1560 chest radiographs from 455 patients with acute hypoxaemic respiratory failure, a CNN could detect ARDS with an area under the receiver operator characteristics curve (AUROC) of 0·92 (95% CI 0·89–0·94). In the subgroup of 413 images reviewed by at least six physicians, its AUROC was 0·93 (95% CI 0·88–0·96), sensitivity 83·0% (95% CI 74·0–91·1), and specificity 88·3% (95% CI 83·1–92·8). Among images with zero of six ARDS annotations (n=155), the median CNN probability was 11%, with six (4%) assigned a probability above 50%. Among images with six of six ARDS annotations (n=27), the median CNN probability was 91%, with two (7%) assigned a probability below 50%. In an external cohort of 958 chest radiographs from 431 patients with sepsis, the AUROC was 0·88 (95% CI 0·85–0·91). When radiographs annotated as equivocal were excluded, the AUROC was 0·93 (0·92–0·95).

**Interpretation:**

A CNN can be trained to achieve expert physician-level performance in ARDS detection on chest radiographs. Further research is needed to evaluate the use of these algorithms to support real-time identification of ARDS patients to ensure fidelity with evidence-based care or to support ongoing ARDS research.

**Funding:**

National Institutes of Health, Department of Defense, and Department of Veterans Affairs.

## Introduction

Acute respiratory distress syndrome (ARDS) is a common critical illness syndrome characterised by the acute onset of severe hypoxaemia and lung oedema of non-cardiac cause in patients with conditions such as sepsis, pneumonia, or trauma. Despite research investment, current treatment for ARDS remains largely supportive and mortality remains at 35%.^1^ Patients who develop ARDS often go unrecognised and do not receive evidence-based care.^2,3^ Bilateral airspace disease on chest radiograph is not only a key criterion in the definition of ARDS, but also a major driver of the definition’s lower reliability.^4^ Intensivists in clinical practice have poor agreement when identifying ARDS findings on chest radiographs (κ 0·13),^5^ and ARDS clinical research study coordinators do little better (κ 0·27).^6^ New approaches for identifying ARDS findings on chest radiographs are needed to support ARDS care.

Deep convolutional neural networks (CNNs) are powerful algorithms that can be trained to recognise findings on visual images. These algorithms have shown physician-level performance across a wide range of medical problems.^7–9^ However, deep learning models must learn millions of parameters; thus training requires large datasets of annotated images.^10^ Generating large datasets for many medical problems can be challenging because clinical data might not be annotated for the finding of interest during routine care. For example, radiologists might not explicitly state whether chest radiographs are consistent with ARDS in their dictated reports. Therefore, conventionally training a CNN to detect ARDS would first require expert physicians to annotate large chest radiograph dataset outside of routine practice, representing a substantial barrier.

Transfer learning is a machine-learning approach where knowledge gained from one problem can be used to help solve related problems.^11^ This approach is particularly applicable to problems where there is only a small amount of data available to train a machine-learning model, but related problems have much larger amounts of available data. When training a CNN to detect ARDS, we hypothesised that if the network could first learn to extract general features from chest radiographs by pretraining the CNN to identify other common findings on chest radiographic studies, it might be able to borrow many of these features, reducing the number of annotated images necessary to train the network to detect findings of ARDS.

In this study, we trained a CNN with 121 layers and 7 million parameters to identify bilateral airspace disease on chest radiographs consistent with ARDS.^12^ We used transfer learning by first pretraining the network on 595 506 radiographs from two centres labelled for common descriptive chest findings (eg, opacity, effusion), but not ARDS. We then trained the network on 8073 radiographs annotated for ARDS. We tested the resulting network on an internal and external test set to evaluate its generalisation performance.

## Methods

### Overview

We trained a CNN with a 121-layer dense neural network architecture (DenseNet)^10^ to detect ARDS on chest radiographs using various transfer learning approaches. An overview of the study design, datasets, and transfer learning approaches are illustrated in the appendix (p 2). The study was approved by the University of Michigan (Ann Arbor, MI, USA) institutional review board with a waiver of informed consent from study participants.

### Datasets

The pretraining dataset combined two publicly available chest radiograph datasets, CheXpert^13^ and MIMIC-CXR.^14^ These images were previously annotated for the presence of any of 14 common clinical findings that can be seen on chest radiographic studies, but not ARDS, using a natural language processing algorithm applied to their associated reports written by radiologists (additional description in the appendix p 3).

The training dataset included all consecutive patients admitted to hospital at the University of Michigan between Jan 1, 2016, and June 30, 2017, who developed acute hypoxaemic respiratory failure, defined as a PaO_2_/FiO_2_ less than 300 while on one of the following respiratory support modalities: invasive mechanical ventilation, non-invasive ventilation, or heated high-flow nasal cannula. Patients received care in the medical, surgical, cardiac, or neurological intensive care unit. Patients transferred to the University of Michigan from outside hospitals were excluded because ARDS might have developed before transfer in these patients. The training dataset was further randomly split by patient, such that 80% was used for CNN training and 20% for validation.

All chest radiographs done during the first 7 days of hospitalisation were used for training. Each chest radiograph was independently reviewed for the presence of ARDS by at least two physicians trained in critical care medicine with an interest in ARDS research. While also reviewing other clinical data from each patient’s hospitalisation, physicians rated whether each image had bilateral opacities present that were consistent with ARDS on a 1–8 ordinal scale. The scale ranged from 1 (no ARDS, high confidence) to 8 (ARDS, high confidence; appendix p 5). We used an eight-point scale to maximise annotation reliability.^15^ The eight-point scale did not have a middle value, which forced annotators to choose whether a radiograph was consistent with ARDS, while still quantifying their uncertainty. The intraclass correlation among physicians reviewing the same image was 0·56.

The internal testing dataset included all consecutive patients admitted to hospital at University of Michigan between July 1 and Dec 1, 2017, who developed acute hypoxaemic respiratory failure, defined as mentioned earlier. There was no patient overlap in the internal training and test sets. Inclusion and exclusion criteria were the same as the training dataset except only chest radiographs obtained when patients had acute hypoxaemic respiratory failure were included to maximise the clinical relevance of the evaluation. Some physicians annotated chest radiographs in both the internal training and test sets. A two class latent class model (ARDS or not ARDS) was used to combine annotations among physicians and determine radiograph labels. A three class model was also explored (ARDS, uncertain, not ARDS). Nine physicians participated in reviewing a subset of 413 chest radiographs in the internal test set, in which each image was reviewed by at least six physicians, including a chest radiologist (appendix p 4).

The external testing dataset included patients admitted to the Hospital of the University of Pennsylvania (Philadelphia, PA, USA) between Jan 1, 2015, and Dec 31, 2017, who were enrolled in a prospective sepsis cohort study (appendix p 6).^16,17^ The dataset included chest radiographs done during the first 5 days of intensive care unit admission, which were previously annotated for ARDS as part of the prospective study (although differently than the University of Michigan datasets). Individual pulmonologist physicians from the University of Pennsylvania who were trained in ARDS clinical research annotated the images as ARDS, equivocal, and not ARDS.^18^ Physicians annotated images as equivocal if the image was deemed difficult to classify due to other abnormalities present on the image or poor technique.^18^ For all datasets, physician reviewers were masked to the CNN result.

### CNN training

The training pipeline and various transfer learning approaches evaluated are illustrated in the appendix (p 2). Code used to train the CNN was adapted from a publicly available source (aligholami/CheXpert-Keras).^19^ First, CNNs were trained to detect 14 common descriptive chest radiograph findings (eg, oedema, infiltrate, and pleural effusion) on the pretraining dataset. Next, the network parameters were fine-tuned to detect ARDS on the University of Michigan training dataset. Networks trained using various transfer learning approaches were compared, which limited the number of network parameters that could be fine-tuned in different parts of the model. CNNs without the pretraining step were also trained. In total, seven CNNs were trained. Additional technical details are described in the appendix (p 7). To improve calibration, Platt scaling was done on the CNN output using parameters derived from the validation portion of the training dataset (appendix p 9).^20^

### Statistical analysis

All seven CNNs trained during the study were evaluated in the University of Michigan internal test set for their ability to discriminate chest radiographs that were consistent with ARDS. The CNN with the highest area under the receiver operator characteristics curve (AUROC) was selected for further evaluation. All subsequent analysis, including external testing, was done solely using this CNN. The area under the precision-recall curve (AUPRC), a measure of the trade-off between sensitivity and positive predictive value, was also used as a secondary metric of discrimination. CNN sensitivity and specificity were calculated after setting the CNN’s calibrated probability threshold to 0·5 for identifying chest radiographs consistent with ARDS. CI generation and statistical testing were done using block bootstrapping to handle repeated measures by resampling at the patient level (appendix p 9).^21^

To compare the best performing CNN to individual physicians, a subset of 413 chest radiographs in the internal test set were reviewed by additional physicians. Nine physicians annotated chest radiographs in this subset, with each reviewing at least 120 images. Individual physician performance was determined by comparing the physician’s annotation to a reference standard derived from the average of five other physicians from this group of nine who reviewed the same image. Individual physician true positive rates (sensitivity) and false positive rates (1-specificity) were plotted against the CNN’s receiver operator characteristics curve on the same patient population. Individual physician precision (positive predictive value) and recall (sensitivity) were plotted against the model’s precision-recall curve.

A boxplot of the CNN’s ARDS probability estimates for each image was created after grouping chest radiographs based on how many physicians annotated the image as ARDS. Gradient-weighted class activation mapping (Grad-CAM) was used to visualise areas of CNN focus within each image when the CNN classified images as ARDS.^22^ After grouping images on the basis of their number of ARDS annotations, Grad-CAM visualisations were used to inspect images assigned the highest ARDS probability to gain insight into CNN decisions.

As a secondary analysis in the University of Michigan test set, CNN performance was compared in patients defined based on demographic subgroups (age, sex, race, and body-mass index [BMI]). CNN calibration was also assessed by generating a calibration plot and determining the intercept and slope (appendix p 12). Because physicians annotating the University of Michigan dataset also identified the time when patients met all ARDS criteria, the time from ARDS onset to CNN detection was quantified to determine if potential delays might occur if the network was deployed in practice. Delay could occur if the CNN did not identify ARDS on the first chest radiograph that physicians identified as ARDS, but on a subsequent chest radiograph. Additionally, an exploratory analysis was done to evaluate the CNN performance using the three-class latent model to categorise chest radiographs (appendix p 11).

In the external test set, chest radiographs had been previously annotated ARDS, equivocal, or not ARDS. To evaluate the best performing CNN in this dataset, both chest radiographs annotated as equivocal or not ARDS were analysed as not ARDS. In a secondary analysis, performance metrics were calculated after chest radiographs labelled equivocal were excluded. A boxplot of ARDS probabilities was created grouping chest radiographs based on these annotation categories.

### Role of the funding source

The funders had no role in the study design; collection, analysis, and interpretation of data; writing of the report; or the decision to submit the Article for publication.

## Results

Demographics of each dataset are reported in the table. The University of Michigan training set included 8072 chest radiographs from 1778 patients, with 2665 (33%) consistent with ARDS based on physician review. The University of Michigan internal test set included 1560 chest radiographs from 455 patients, with 438 (28%) consistent with ARDS. The external test set included 958 chest radiographs from 431 patients, with 445 (46%) consistent with ARDS based on physician review. The most common ARDS risk factors were pneumonia followed by sepsis from a non-pulmonary source.

The training, validation, and internal testing AUROCs for all seven CNNs are reported in the appendix (p 10). The best performing CNN (AUROC 0·92, 95% CI 0·89–0·94) had the last convolutional block and subsequent layers fine-tuned to detect ARDS while all others kept fixed after pretraining (appendix p 2; version ii). This CNN had a slightly higher AUROC than a network with all parameters fine-tuned (AUROC 0·91, 95% CI 0·89–0·94; appendix p 2; version iii), a difference that was not statistically significant (p=0·56). However, this later network had a large drop between training and validation performance (AUROC 0·97–0·89; p<0·001), suggesting overfitting (appendix p 10). CNNs that did not undergo chest radiograph pretraining had lower performance (appendix p 10). Therefore, all subsequent analysis was done using the CNN illustrated in the appendix (p 2; version ii).

**Figure 1:**
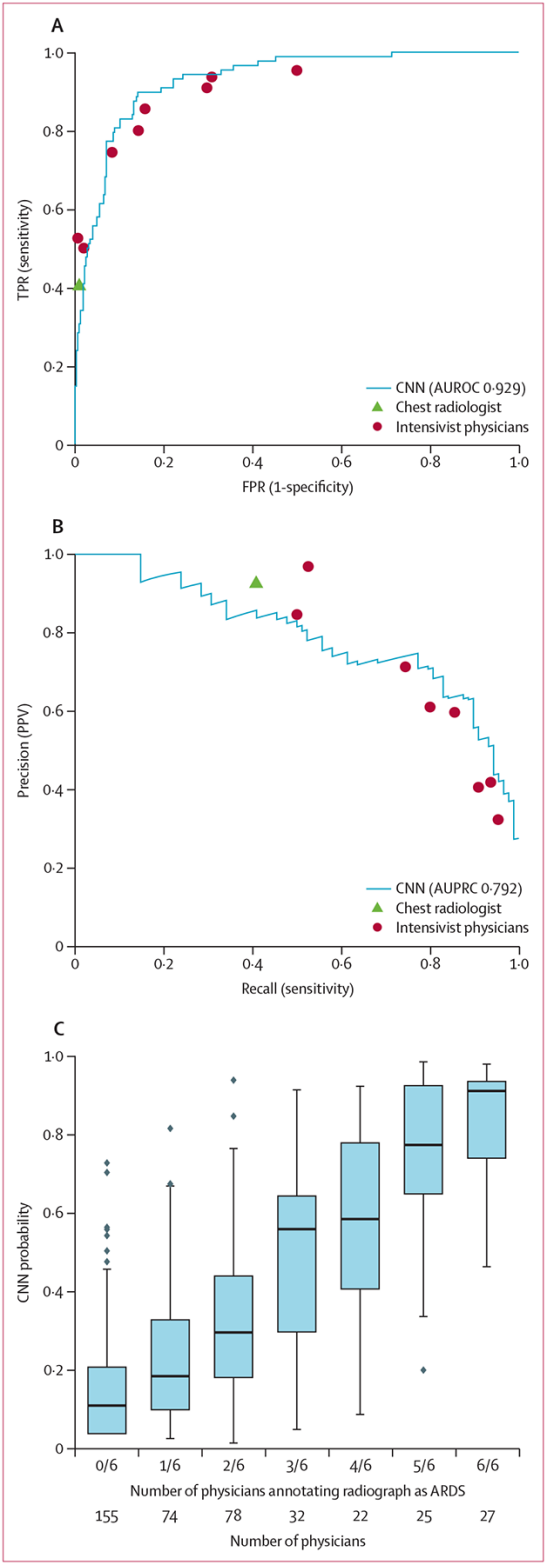
CNN performance for identifying ARDS on chest radiographs compared to individual physician performance in the internal holdout test set The deep CNN was compared with individual physicians in the subgroup of 413 chest radiographs that were each reviewed by at least six physicians, including a chest radiologist and physicians trained in intensive care medicine. Individual physician performance was determined using a reference standard that was derived based on ARDS annotations from the five other physicians reviewing the same radiograph. (A) CNN receiver operating characteristics curve plotted against individual physician TPR and FPR, and AUROC. (B) CNN precision-recall curve plotted against individual physician precision (PPV) and recall (sensitivity), and AUPRC. (C) CNN probability outputs for chest radiographs grouped by the number of physicians annotating each as ARDS. Boxplots show median, 25th and 75th percentile, and 1·5 × IQR. Dots represent points outside this range. CNN=convolutional neural network. ARDS=acute respiratory distress syndrome. AUROC=area under the receiver operator characteristic curve. AUPRC=area under the precision-recall curve. TPR=true positive rate. FPR=false positive rate. PPV=positive predictive value.

The CNN was evaluated in a subset of 413 chest radiographs from the University of Michigan internal test set with additional physician reviews to enable comparisons to individual physicians (figure 1). When evaluated against this stronger reference standard, the AUROC was 0·93 (95% CI 0·88–0·96). CNN sensitivity was 83·0% (95% CI 74·0–91·1) and specificity was 88·3% (95% CI 83·1–92·8) using a threshold of 50% probability to identify ARDS. The AUPRC was 0·79 (95% CI 0·63–0·88). Using the chest radiologist alone as the reference standard, the AUROC was 0·92 (95% CI 0·87–0·96). Compared with each physician, the CNN showed similar performance, with physicians tracking along the CNN’s receiver operator characteristic curve, at either higher specificity and lower sensitivity, or vice versa.

The ARDS probability estimated by the CNN also tracked with the number of physicians who annotated the radiograph as consistent with ARDS (figure 1). Among chest radiographs with zero of six ARDS annotations (n=155), the median calibrated CNN probability was 11% with six (4%) of 155 assigned a probability above 50%. Among chest radiographs with six of six ARDS annotations (n=27), the median CNN probability was 91% and with two (7%) of 27 assigned a probability below 50%. Among chest radiographs with disagreement among physicians (eg, three of six physicians annotating the radiograph as ARDS), the CNN assigned intermediate probabilities.

**Figure 2:**
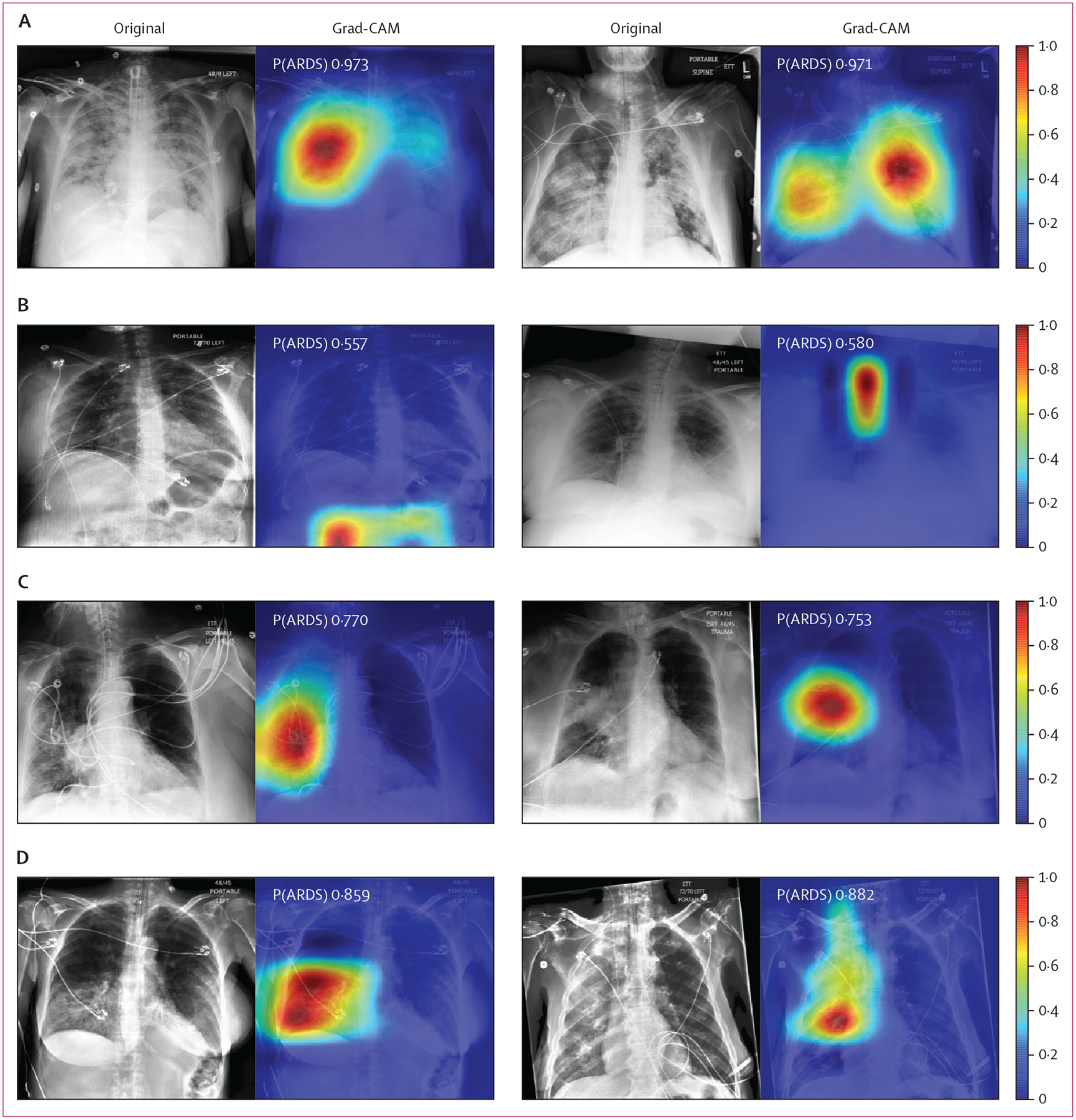
Visualising CNN activations in chest radiographs for error analysis in ARDS detection Chest radiographs were grouped based on CNN probabilities of ARDS and physician ARDS annotations and then Grad-CAM was used to localise areas used by the CNN to identify ARDS within the radiographs. The heat map illustrates the importance of local areas within the image for classification. The importance value is scaled between 0 and 1 where a higher number indicates that the area is of higher importance for classifying the image as consistent with ARDS. (A) Chest radiographs annotated as ARDS by six of six physicians and assigned a high CNN probability. (B) Chest radiographs scored as consistent by six of six physicians but assigned a lower probability by the CNN. (C) Chest radiographs annotated as ARDS by zero of six physicians but assigned a high probability by the CNN. (D) Chest radiographs with disagreement among physicians (three of six physicians annotating ARDS) and assigned a high probability by the CNN. CNN=convolutional neural network. ARDS=acute respiratory distress syndrome. Grad-CAM=gradient-weighted class activation mapping. P(ARDS)=probability that the chest radiograph is consistent with ARDS.

Among chest radiographs correctly classified (six of six physician annotations for ARDS, high CNN probability), the CNN focused on regions of the lung that exhibited opacities based on Grad-CAM visualisations (figure 2). Among chest radiographs annotated by all six physicians as ARDS, but assigned lower CNN probabilities, the CNN focused on findings outside the lung. Among chest radiographs without ARDS annotations, but assigned a higher CNN probability, the CNN focused on right-sided unilateral disease. Finally, among radiographs with disagreement among clinicians, but assigned a higher CNN probability, the CNN focused on the right lung, which appeared to have more prominent disease.

**Figure 3:**
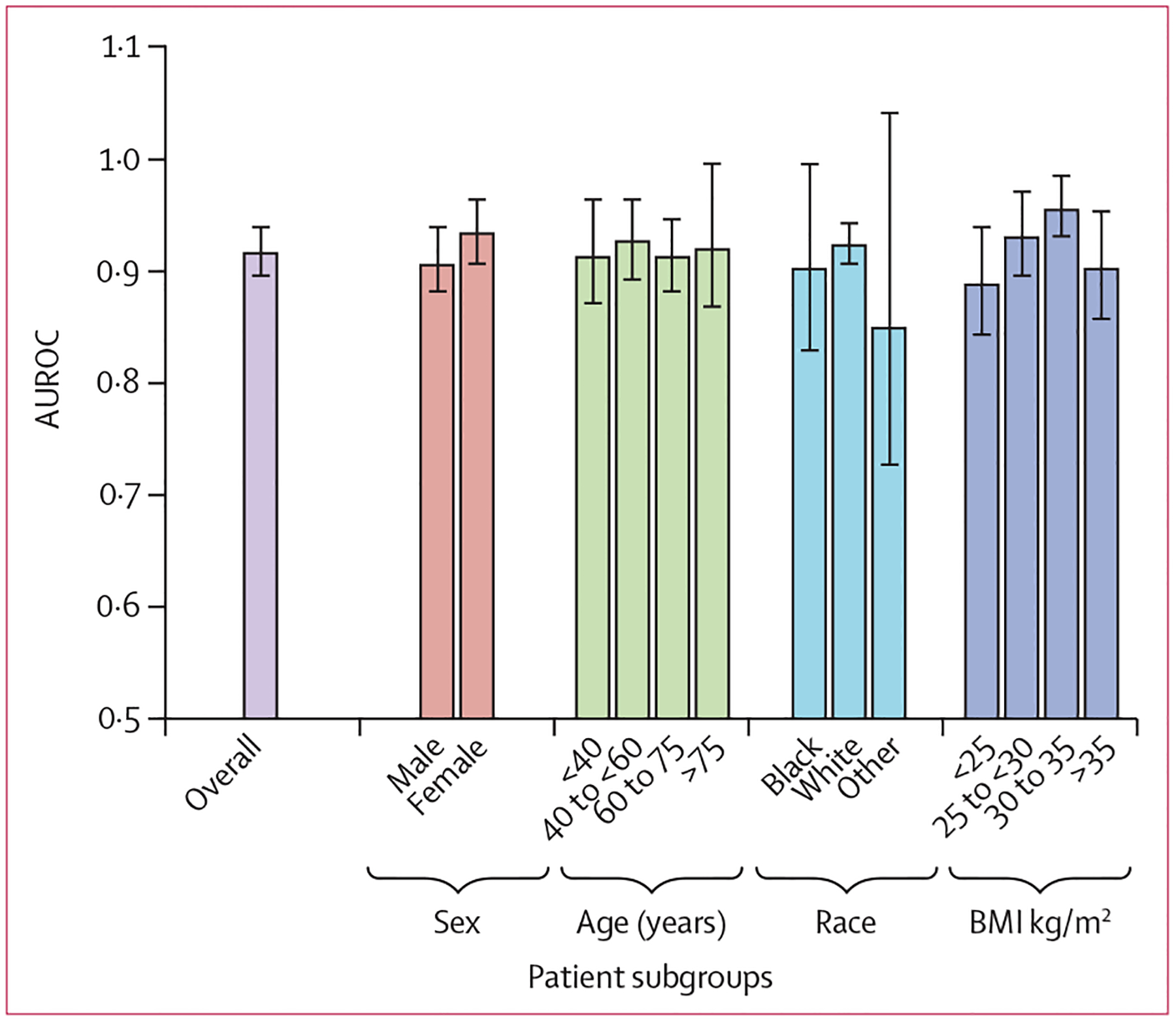
CNN performance for identifying ARDS on chest radiographs by patient subgroups Race categories were self-reported. Error bars represent 95% CI estimates of the AUROC. Race category other includes patients who are Asian, American Indian, Native Alaskan, Native Hawaiian, other Pacific Islander, or unknown race. CNN=convolutional neural network. ARDS=acute respiratory distress syndrome. AUROC=area under the receiver operator characteristics curve. BMI=body-mass index.

CNN performance was evaluated on demographic subgroups as a secondary analysis using the entire University of Michigan internal test set (figure 3; appendix p 10). AUROC was not significantly different in men (0·91) and women (0·93; p=0·21). AUROC was also not significantly different in White patients (0·92) and Black patients (0·90; p=0·63). It was also not significantly different across age categories. The AUROC was higher in patients with a BMI of 30–35 kg/m^2^ (0·96), compared with a BMI of less than 25 kg/m^2^ (0·89; p=0·004) and BMI of more than 35 kg/m^2^ (0·96 *vs* 0·90; p=0·026). CNN calibration is reported in the appendix (p 11). In an analysis to determine if there would be detection delay in patients the CNN correctly identified as having ARDS, the median time when the model detected ARDS was 0 h after physician reviewers determined the patient met ARDS criteria, with an IQR of 4 h before 0 h after onset. The median time between the first and last chest radiograph for any individual patient was 19 h. An exploratory analysis evaluating CNN performance after categorising chest radiographs into three groups, which found that the CNN had very high performance when chest radiographs assigned to the uncertain class were excluded, is presented in the appendix (p 11).

**Figure 4:**
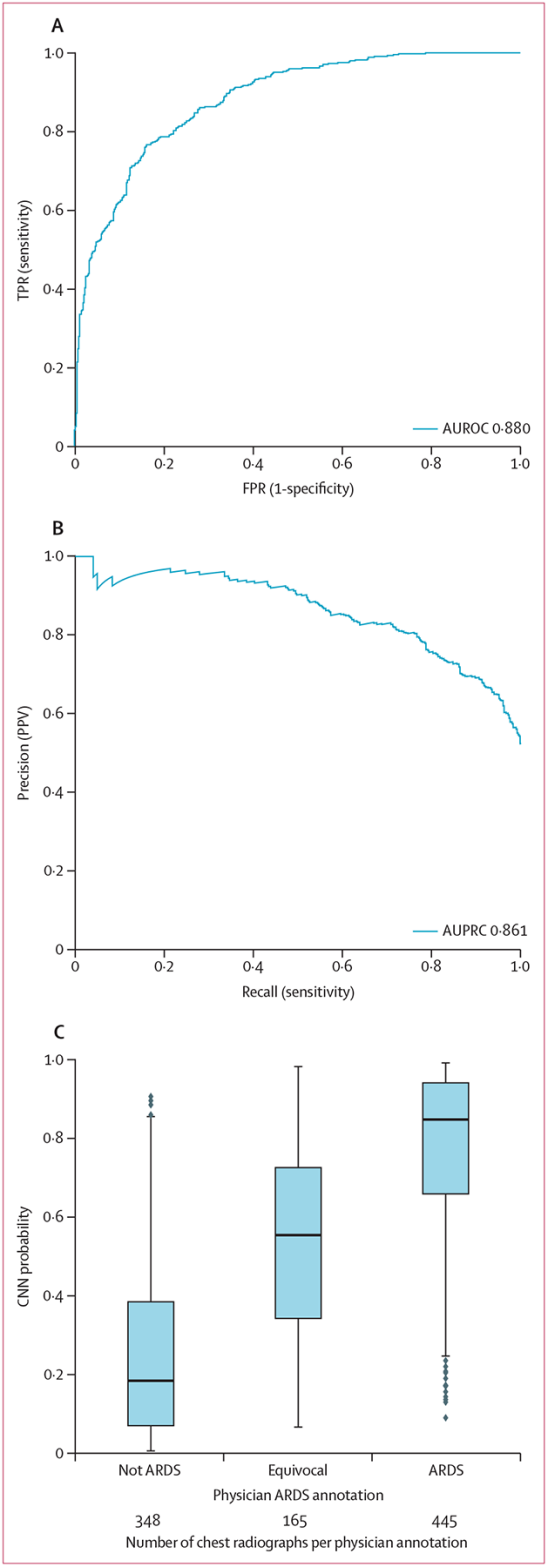
CNN performance for identifying ARDS on chest radiographs in an external test set Receiver operator curve which is the plot of the TPR and FPR (A), and precision-recall curve which is the plot of the PPV and the model sensitivity (B), and probability outputs from the CNN across chest radiograph annotation categories, showing median, 25th and 75th percentile, and 1·5 × IQR (C). Dots represent points outside this range. CNN=convolutional neural network. ARDS=acute respiratory distress syndrome. AUROC=area under the receiver operator characteristic curve. AUPRC=area under the precision-recall curve. TPR=true positive rate. FPR=false positive rate. PPV=positive predictive value.

The CNN was then evaluated on the University of Pennsylvania external test set. The network’s AUROC was 0·88 (95% CI 0·85–0·91) and AUPRC was 0·86 (95% CI 0·82–0·90; figure 4). When chest radiographs annotated as equivocal were excluded as a secondary analysis, the AUROC was 0·93 (95% CI 0·92–0·95) and AUPRC was 0·95 (95% CI 0·92–0·96). The CNN assigned intermediate probabilities to chest radiographs annotated as equivocal compared with chest radiographs annotated as ARDS and not ARDS. CNN calibration was not substantially worse than in the internal test set (appendix p 12).

## Discussion

ARDS is a common critical illness syndrome, which can be difficult to consistently identify due to variation in interpretation of chest imaging. We trained a CNN to detect ARDS on chest radiographs that showed performance equivalent to physicians involved in ARDS research and generalised to chest radiographs from an external centre.

The increasing availability of digitally archived medical imaging datasets have catalysed many efforts to develop machine-learning models to support patient care. However, the insufficiency of annotations relevant to important clinical problems remains a major impediment.^23^ Given the data-hungry nature of these CNN-based machine-learning models, investigators are left choosing to do costly and time-consuming annotation by human experts or use an imperfect labelling approach. We used a hybrid approach, leveraging a dataset of chest radiographs carefully annotated for ARDS and two large datasets for common chest radiographic findings. Using transfer learning, these large datasets enabled the CNN to learn a general representation of chest radiographs to jump-start ARDS training.^11^ Without this transfer learning approach, the CNN was unable to achieve similar ARDS accuracy.

We found the CNN trained to detect ARDS had only a small decline when applied to chest radiographs at an external centre. This finding is in contrast to other work training CNNs to detect pneumonia, where a network trained using data from two institutions failed to generalise to a third.^24^ Investigators postulated that the network learned confounded features—eg, features of an image distinguishing the hospital system where it was acquired to determine if the image was consistent with pneumonia. The constraints set on our network during transfer learning, potentially limiting its ability to overfit, might be one potential explanation for our network’s preserved performance. Other important differences include the use of a two-centre pretraining dataset and expert physician annotations, rather than radiology reports, to evaluate performance.

We generated class activation maps (Grad-CAM) to see where the CNN was focusing when classifying radiographs as ARDS. When classifying images correctly, the CNN appeared to focus on abnormalities within the lungs. When the CNN misclassified images, it sometimes appeared to identify unilateral lung disease or findings outside the lungs. CNNs have been suggested to make decisions on the basis of the presence of small local features within images, with less emphasis placed on their spatial ordering.^25^ Therefore, CNNs might have difficulty learning that local textural features with the appearance of lung injury must be within the lungs to represent ARDS. Networks that better account for these relationships—eg, through jointly segmenting the lungs followed by classifying abnormalities—might lead to improved performance.

ARDS is often under-recognised or identification is delayed in clinical practice, and patients do not always receive guideline-recommended interventions,^1^ including lung protective mechanical ventilation and prone positioning.^2,3^ Automated alert systems have been proposed to improve ARDS identification,^26^ because when physicians recognise that patients have ARDS, they are more likely to provide evidence-based interventions.^2^ Previous efforts to develop automated ARDS detection systems typically analyse the electronic health records and the text of radiology reports.^27^ In contrast, the CNN developed here analyses digital chest radiographs directly. However, deploying the network might require additional health technology investment, including automated identification of patients with a PaO_2_/FiO_2_ of less than 300 from electronic health records, computational analysis of radiographs stored in picture archiving and communications systems, and a mechanism for providing the results to physicians.

Our study has limitations. First, ARDS is a syndrome defined by a shared set of clinical features, and the diagnosis of ARDS is currently based on the combination of clinical and radiological criteria for which there is no established or easily available gold standard diagnostic test.^28^ Therefore, training a CNN to detect findings of ARDS relied on annotations from expert physicians, who also have imperfect reliability.^4^ To address this issue, we used a standardised scale, and reference standard, that combined multiple independent physician reviews to improve reliability. Second, the external test set was annotated using an alternative method useful in ARDS translational research, but perhaps less optimal for algorithm evaluation and did not include exact time-stamps of ARDS onset, preventing an assessment of possible detection delay. Nonetheless, the strong performance in the external dataset, even with a different reference standard and different physician annotators, suggests that the network is robust. Third, selection bias among patients in the datasets used for training could limit its generalisability. While the distribution of patients analysed is typical of other ARDS research,^2^ it did not have balance between men and women, and certain patients had lower representation (eg, trauma). Thus, validation of the network in additional patient populations and clinical settings should be done. Finally, although we evaluated the network after setting a threshold of 50% probability to identify ARDS, the threshold ultimately used to determine whether a patient has ARDS is probably context specific. In some scenarios (eg, provision of lung protective ventilation), a lower threshold to maximise sensitivity is preferable, whereas in other scenarios (eg, recruitment of patients to ARDS clinical trials), a higher threshold to maximise specificity might be preferred.

In summary, these results show the power of deep learning models, which can be trained to accurately identify chest radiographs consistent with ARDS. Further research is needed to evaluate how the use of these algorithms could support real-time identification of ARDS patients to ensure fidelity with evidence-based care or to support ongoing ARDS research.

## Supplementary Material

1

## Figures and Tables

**Table:** Characteristics of patients in training and testing datasets

	Training dataset (University of Michigan)	Internal testing dataset (University of Michigan)	External testing dataset (University of Pennsylvania)
Patients	1778	455	431
Chest radiographs	8072	1560	958
Radiographs with ARDS*	2665 (33%)	438 (28%)	445 (46%)
Age, years	62 (51–71)	63 (53–72)	61 (52–69)
Sex			
Male	1036 (58%)	266 (58%)	251 (58%)
Female	742 (42%)	189 (42%)	180 (42%)
Race			
White	1515 (85%)	377 (83%)	273 (63%)
Black	164 (9%)	49 (11%)	129 (30%)
Other or unknown†	99 (6%)	29 (6%)	29 (7%)
ARDS risk factor			
Pneumonia	591 (33%)	126 (28%)	101 (23%)
Aspiration	215 (12%)	39 (9%)	NA
Non-pulmonary sepsis	394 (22%)	114 (25%)	330 (77%)
Trauma	110 (6%)	28 (6%)	NA
APACHE score	67 (52–85)	68 (55–86)	98 (72–129)
30-day mortality	420 (24%)	119 (26%)	188 (44%)

Data are n, n (%), or median (IQR). ARDS=acute respiratory distress syndrome. NA=not applicable. APACHE=Acute Physiology and Chronic Health Evaluation.

* Based on physician reviews.

† Includes Asian, American Indian, Native Alaskan, Native Hawaiian, or other Pacific Islander.

## Research in context

### Evidence before this study

Acute respiratory distress syndrome (ARDS) is a common critical illness syndrome associated with significant mortality, but is under-recognised in clinical practice. A key criterion of the Berlin ARDS definition is the presence of bilateral airspace disease on chest imaging, but physician interpretation of chest imaging studies for this finding is highly variable. Machine-learning algorithms called deep convolutional neural networks have been trained to automatically identify many relevant findings on images with expert physician-level accuracy (eg, diabetic retinopathy and skin cancer). We were unaware of their use to analyse chest radiographs for ARDS. We searched PubMed for studies indexed in MEDLINE using the key words and MeSH terms, (“ARDS” OR “Acute Respiratory Distress Syndrome” OR “Respiratory Distress Syndrome, Adult” [MeSH Terms]) AND (“Deep Learning” [MeSH Terms] OR “Convolutional Neural Network”). We did not identify any studies that applied these algorithms to chest radiographs for ARDS in this search. Because ARDS can also be seen in patients with COVID-19, we also searched for studies specifically related to COVID-19 using the keywords and MESH terms, (“COVID-19” AND “Deep Learning” [MeSH Terms]). We identified many studies using deep learning methods to diagnosis COVID-19 using chest radiographs and a few designed to determine COVID-19 severity, but none were specifically designed to detect findings of ARDS.

### Added value of this study

We successfully trained a deep convolutional neural network to analyse chest radiographs and identify findings consistent with ARDS. We found that the resulting algorithm could identify ARDS findings with high accuracy in patients from two centres. Visual evaluation of the algorithm outputs confirmed that it learned to focus on regions of the lung that exhibited opacities when classifying images as consistent with ARDS. Performance of the algorithm was consistent with or higher than individual physician performance.

### Implications of all the available evidence

These results demonstrate the power of machine-learning algorithms in the analysis of chest radiographs for ARDS. Further research is needed to evaluate the use of such algorithms to support management of ARDS patients in clinical practice or to more consistently recruit ARDS patients into clinical studies.
