# Supplementary material for: Deep learning to detect acute respiratory distress syndrome on chest radiographs: a retrospective study with external validation
Source: Lancet Digit Health. Author manuscript; Available in PMC 2021 Jun 7. (PMC8182690; doi:10.1016/S2589-7500(21)00056-X)
Supplement: 1 [file NIHMS1708195-supplement-1.pdf]

THE LANCET
Digital Health

Supplementary appendix

This appendix formed part of the original submission and has been peer reviewed. We post it as supplied by the authors.

Supplement to: Sjoding MW, Taylor D, Motyka J, et al. Deep learning to detect acute respiratory distress syndrome on chest radiographs: a retrospective study with external validation. *Lancet Digit Health* 2021; published online April 20. https://doi.org/10.1016/S2589-7500(21)00056-X.

Supplementary Appendix

Deep Learning to Detect Findings of the Acute Respiratory Distress Syndrome on Chest Radiographs

Supplementary Methods……………………………………………………………………………2

Supplementary Figure 1. Study design, CNN architecture, transfer learning approaches……..2

Supplementary Table 1: Chest radiograph annotation scale for the UM cohort…………………5

Supplementary Table 2: Fit Statistics for the Latent Class Models ………………………………6

Supplementary Results……………………………………………………………………………10

Supplementary Table 3: Training, validation, and test performance of all 7 CNNs in the UM datasets………………………………………………………………………………………10

Supplementary Table 4: CNN performance on patient subgroups in the UM test set………11

Supplementary Table 5. CNN performance in the UM test dataset based on the 3-class latent class model……………………………………………………………………………..11

Supplementary Figure 2. Calibration of the CNN in the UM internal test set………………...12

Supplementary Figure 3. Calibration of the CNN in the UPenn external test set……………12

References…………………………………………………………………………………….13

Supplementary Figure 1. Study design, CNN architecture, transfer learning approaches

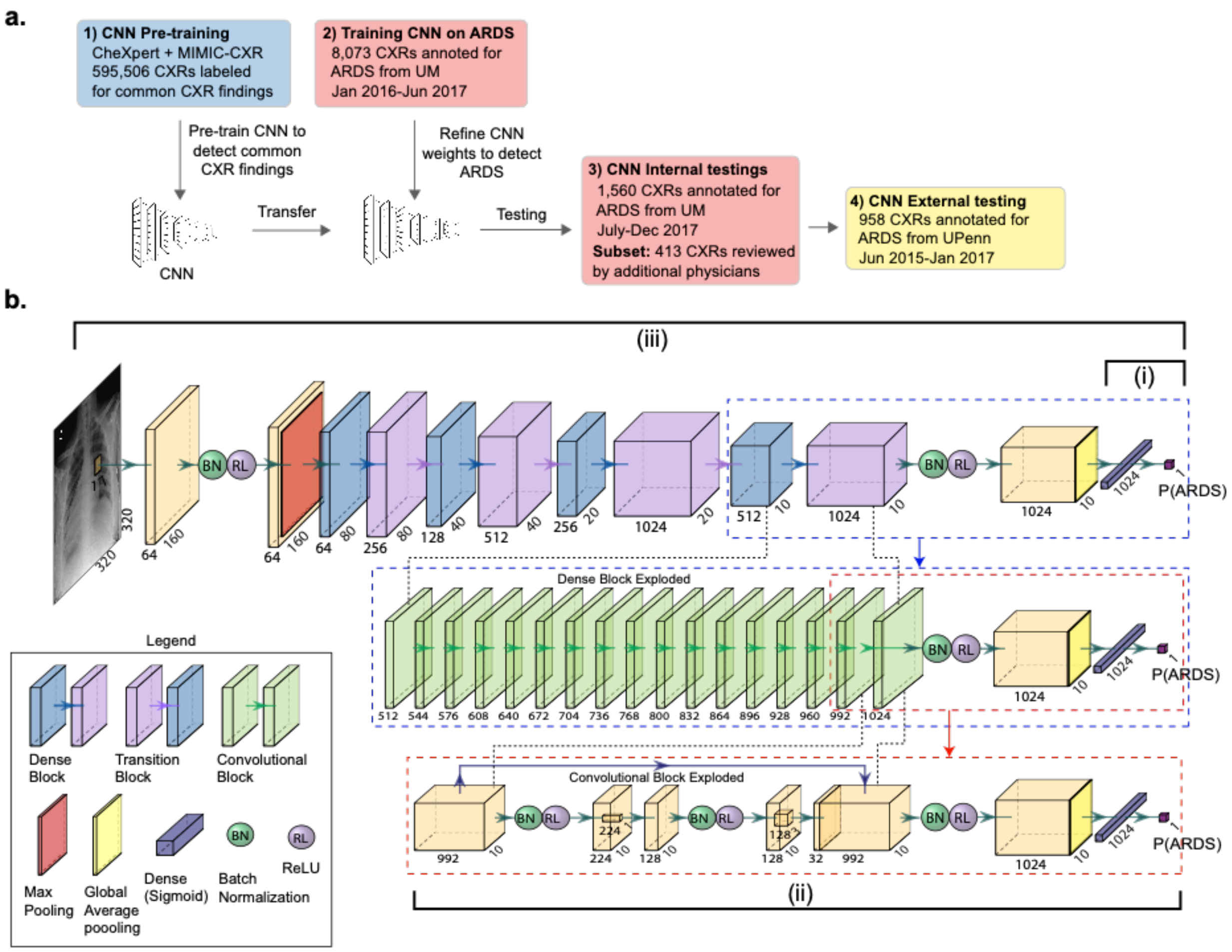

A. Study overview: 1) Deep convolutional neural networks (CNNs) were first pre-trained to detect common chest radiographic findings (e.g. infiltrate, pleural effusion) using 595,506 chest radiographs (CXRs). 2) CNN weights were then refined to detect ARDS using the UM training set of 8,073 chest radiographs annotated for ARDS. 3) Once training and validation was completed, CNNs were tested on a separate internal testing dataset from UM, including a subset by additional physicians. 4) The best performing CNN was also tested on an external test set of patients from UPenn. **B**. A 121-layer CNN with a dense network architecture was trained to identify ARDS. The CNN transforms a 320x320 pixel chest radiograph over multiple layers into increasingly abstract representations that are used to estimate the probability of ARDS (P(ARDS)). Transfer learning approaches that minimized the number of network parameters trained on the smaller dataset annotated for ARDS were evaluated: (i) refining parameters in the final layers (3,073 total parameters retrained), while keeping all other layers fixed after pre-training; (ii) refining parameters in the last convolutional block and subsequent layers (169,153 parameters retrained); (iii) refining all parameters on the ARDS dataset after pre-training (6,954,881 parameters retrained). The CNN illustrated is not to scale and zero padding layers are not shown.

Additional description of the chest radiograph pre-training dataset

The chest radiograph pre-training dataset was created using two publicly available datasets, CheXpert (V1.0)[1] and MIMIC-CXR (V1.0).[2] CheXpert includes 224,316 chest radiograph studies from 65,240 patients performed in inpatient and outpatient centers at Stanford Hospital between October 2002 and July 2017. MIMIC-CXR includes 227,835 chest radiograph studies from 65,379 patients who presented to the emergency department between 2011 and 2016 at Beth Israel Deaconess Medical Center. Images had been previously annotated for the presence of any of 14 common clinical findings but not ARDS (enlarged cardiomediastinum, cardiomegaly, airspace opacity, lung lesion, edema, consolidation, pneumonia, atelectasis, pneumothorax, pleural effusion, pleural other, fracture, support device, no finding) using a natural language processing algorithm applied to their associated reports written by radiologists. Images were previously converted from Digital Imaging and Communications in Medicine (DICOM) format to 320 by 320 pixel, 8-bit grayscale JPEG images, and histogram equalization was applied.

Additional description of the UM datasets

The University of Michigan (UM) internal training dataset was created from chest radiographs performed during the first 7 days on consecutive patients hospitalized with acute hypoxemic respiratory failure between January 2016 to June 2017 at UM. The UM internal testing dataset was created from patients hospitalized with acute hypoxemic respiratory failure between July to December 2017. There was no overlap among patients in the training and the internal testing datasets. Acute hypoxemic respiratory failure was defined as having a $PaO_2/FiO_2 < 300$ while receiving invasive mechanical ventilation, non-invasive ventilation, or heated high flow nasal cannula during the first 7 days of hospitalization. PaO_2/FiO_2 was calculated using PaO_2 values obtained from arterial blood gas results and the recorded FiO_2 at the time the blood gas was performed. Patients who transferred from other hospitals were excluded because ARDS may have developed prior to transfer and the timing of ARDS could not be adequately determined.

In the UM internal test set, as an additional inclusion criteria, chest radiographs obtained during periods when patients met criteria for acute hypoxemic respiratory failure were analyzed to maximize the clinical relevance of the evaluation. A subset of 413 radiographs from consecutive patients hospitalized between August 15 – October 2, 2017 were each reviewed by additional physicians to compare the CNN performance to individual physician performance. Nine physicians participated in the reviews, with each physician reviewing at least 120 chest x-rays. Among the physicians was a chest radiologist with 5 years of experience who reviewed all radiographs in the subset. The group also included 6 physicians who completed a pulmonary and critical fellowship and were in general practice, 1 physician who completed an emergency department and critical care fellowship and was in general practice, and 1 physician who completed 2 years of a pulmonary and critical care fellowship. All physicians had interest and experience in ARDS research.

Determining chest radiograph class labels in the UM cohorts

Physicians annotated each chest radiographs for ARDS while reviewing all other clinical details about the patient's hospitalization. Additional details about the ARDS review process has been previously published.[3] For each chest radiograph, physicians answered the following question: "Are their bilateral opacities on this chest x-ray that are consistent with ARDS?" and recorded their response using an 8-point ordinal confidence score.

Supplementary Table 1: Chest radiograph annotation scale for the UM cohort

Score	Description
1	No ARDS, high confidence
2	No ARDS, moderate confidence
3	No ARDS, low confidence
4	No ARDS, equivocal confidence
5	Yes ARDS, equivocal confidence
6	Yes ARDS, low confidence
7	Yes ARDS, moderate confidence
8	Yes ARDS, high confidence

An 8-point score was used because the reliability is maximized when clinical scales with 7 or more categories is used.[4] An 8-point scale was used specifically because it did not have a middle value, forcing physicians to choose whether they felt a radiograph was consistent with ARDS, while still quantifying their uncertainty. Inter-rater reliability among physicians reviewing the same chest radiograph was 0.56 as measured by the intra-class correlation, which is considered moderate reliability. Based on the Spearmon-Brown prophecy formula, a gold standard which combed annotations from 5 independent physician reviewers would have a reliability of 0.87, which is considered near perfect reliability. This is the rationale for the additional physician reviews in the UM testing subset.

To determine the class label for each chest radiograph (consistent with ARDS, inconsistent with ARDS) in the University of Michigan datasets, a latent class model with two classes was fit using all annotations performed on each chest radiograph. In this model, the specific physician reviewer annotating each image was modeled as a fixed effect to account for the fact that individual physicians may have varying thresholds for scoring images as consistent with ARDS on the 1-8 scale. After fitting the model, the posterior probability of each class membership was estimated for each radiograph, and the chest radiograph was assigned to the class with the highest probability estimate. A two class model (“ARDS”, “Not ARDS”) was considered for the primary analysis, as this represents how detection of ARDS is performed in clinical practice. We

also explored the CNN results after fitting a three class model (“ARDS”, “equivocal”, “Not ARDS”), to determine how well the CNN performed on chest radiographs that physicians felt had less ambiguity. Average posterior probabilities for each class and fit statistics are presented below.

Supplementary Table 2: Fit Statistics for the Latent Class Models in the UM test dataset

**Two class model**	**Class 1 (“Not ARDS”)**	**Class 2 (“ARDS”)**	
N, chest x-rays	1122	438	
Posterior probability	0.981	0.964	
**Three class model**	**Class 1 (“Not ARDS”)**	**Class 2 (“uncertain”)**	**Class 3 (“ARDS”)**
N, chest x-rays	922	307	331
Posterior probability	0.934	0.790	0.957
**Fit Statistics**	**Two class model**	**Three class model**	
Log likelihood	-11245.106	-11099.393	
AIC	22526.21	22238.79	
BIC	22644.49	22370.21	

Additional description of the UPenn external testing datasets

Chest radiographs in the external test set were from patients enrolled in the “Molecular Epidemiology of Sepsis in the ICU” (MESSI) cohort. Patients were enrolled if they were admitted to the intensive care unit with infection-associated organ failure, and were excluded if an alternative diagnosis explained their systemic inflammatory response syndrome, for declining life support on admission, or for lack of informed consent. For patients enrolled, all chest imaging studies obtained during the first 6 days over the ICU stay were reviewed by a trained physician investigator for the presence of ARDS. In this cohort, chest radiographs were annotated as “ARDS”, “equivocal”, and “not ARDS,” with consensus review performed in approximately 5% of images. Physicians annotated images as “equivocal” if the image was

deemed difficult to classify due to other abnormalities present on the image or poor technique. When evaluating the CNN in this dataset, both chest radiographs annotated as “equivocal” or “not ARDS” were analyzed as “not ARDS.” In a secondary analysis, performance metrics were also calculated after chest radiographs labeled “equivocal” were excluded.

Chest Radiograph pre-processing

Chest radiographs were exported from the Picture Archiving and Communication System (PACS) in Digital Imaging and Communications in Medicine (DICOM) format. Images were converted to 8-bit grayscale JPEG format, histogram equalized, and resized to a target dimension of 320 by 320 such that the smaller dimension was shrunk to the target size and large dimension was squashed to the target size. Chest radiographs from the University of Pennsylvania (UPenn) were exported from the UPenn PACS system in a DICOM format and pre-processed in an identical manner as in the University of Michigan datasets.

Additional Description of Convolutional Neural Network training

All CNNs were trained using Keras (version 2.2.4) with Tensorflow (version 1.13.1) in Python (version 3.6). A 121-layer dense network architecture (DenseNet121) was used.[5] Prior to training the CNN to detect ARDS, networks were either first pre-trained to detect common descriptive findings on chest radiographs (chest radiograph pre-training), initialized using parameter weights from pre-trained natural images from ImageNet (e.g. animals, plants, household objects)[6], or randomly initialized.

CNN pre-training

When pre-training the CNN to detect common chest radiograph findings, 592,540 images were used for training and 2,966 images were used for validation. Training images were further augmented by randomly rotating them up to 15 degrees and translating them up to 10%. During

training, parameter weights were initialized using ImageNet weights. The network had 14 total output nodes with sigmoid activation functions that corresponded to each radiograph finding (described above). The model was trained to minimize the binary cross entropy loss across these activation nodes. Parameters of the model were optimized using Adam with an initial learning rate of 10^{-4}.[7] If the validation set's loss did not improve for 2 consecutive epochs, the learning rate was reduced by a factor of 10. The learning rate could be reduced to a minimum of 10^{-8}. The model was trained for three epochs to minimize computational time. The model from the epoch with the highest validation area under the receiver operator characteristic curve (AUROC) was selected and used in the subsequent ARDS training steps. The validation AUROC was highest after the second epoch.

CNN training to detect ARDS

The same 121-layer dense network architecture was used as in the pre-training steps, but the final layer of the network was changed to a single node with a sigmoid activation function to estimate the probability of ARDS. Similar to the pre-training step, images were augmented by randomly rotating them up to 15 degrees and translating them up to 10%. CNNs were trained for 10 epochs using the same learning rate schedule as the pre-training step. The model from the epoch with the highest validation AUROC was selected as the final model and used for testing.

Transfer learning approaches were evaluated during ARDS training that limited the number of network parameters that were fine-tuned to detect ARDS using the UM training data. Approaches evaluated included: i) refining parameters in the final layers, including a batch normalization (BN), rectified linear unit (ReLU) activation which does not have parameters, average pooling, and dense layer (3,073 total parameters retrained), while keeping all other layers fixed after pre-training; ii) refining parameters in the last convolutional block and

subsequent layers, including a BN, ReLU, convolution, BN, ReLU, convolution, BN, ReLU, average pooling, dense layer (169,153 parameters retrained); iii) refining all parameters on the ARDS dataset after pre-training (6,954,881 parameters retrained).

To improve calibration of the model, the CNN output was transformed using Platt scaling.[8] In Platt scaling, a univariate logistic regression model is fit that uses the CNN output as the independent variable and the binary outcome as the dependent variable. The intercept and slope of the logistic regression model represent the scaling parameters which are then used to transform the CNN outputs within a sigmoid function. These scaling parameters were determined using the validation portion of the training dataset.

Additional description of the statistical analysis

To account for clustering of chest radiographs within patients when determine 95% confidence intervals for all performance metrics, including the area under the receiver operator characteristic curve (AUROC), the area under the precision recall curve (AUPRC), sensitivity, and specificity, we drew 1000 cluster bootstrap samples (drawing samples at the patient-level with replacement). We used a similar bootstrapping procedure when evaluating differences in AUROC.[9] P values < 0.05 was considered statistically significant. All statistical analysis was performed in Stata version 16.1.

*Supplementary Table 3***.** Training, validation, and test performance of all 7 CNNs in the UM datasets

		CNN Performance for detecting ARDS, AUROC (95%CI)		
		**UM Internal training dataset**		**UM internal testing dataset**
**Pre-training Datasets**	**Network layers refined during ARDS training**	**Training sample (N=6,493) Annotations per image = 2.5**	**Validation sample (N=1,578) Annotations per image = 2.5**	**(N=1,560) Annotations per image = 3.5**
Chest x-ray + ImageNet	Last BN and dense layer	0.875 (0.86-0.889)	0.867 (0.838-0.894)	0.907 (0.882-0.931)
	**Last convolutional block and subsequent layers**	**0.888 (0.874-0.901)**	**0.884 (0.858-0.908)**	**0.916 (0.894-0.936)**
	All layers	0.965 (0.96-0.971)	0.893 (0.866-0.917)	0.913 (0.89-0.933)
ImageNet	Last BN and dense layer	0.67 (0.649-0.691)	0.688 (0.655-0.721)	0.692 (0.651-0.729)
	Last convolutional block and subsequent layers	0.743 (0.724-0.76)	0.731 (0.69-0.766)	0.742 (0.700-0.782)
	All layers	0.969 (0.964-0.973)	0.885 (0.859-0.911)	0.891 (0.867-0.914)
None (randomized weights)	All layers	0.820 (0.805-0.836)	0.820 (0.785-0.85)	0.828 (0.795-0.859)

All deep convolutional neural networks (CNNs) had a 121-layer dense network architecture. Networks underwent chest radiograph pre-training, were initialized using ImageNet parameter weights, or randomly initialized. Networks that underwent chest radiograph pre-training were first trained to detect 14 common descriptive findings on chest radiographs (e.g. infiltrate, pleural effusion) using the publicly available CheXpert and MIMIC-CXR datasets. During training on ARDS, parameters in some layers were refined while others kept fixed after pre-training. The internal training and validation datasets included patients hospitalized between January 1, 2016 and June 30, 2017 at a single center. The internal testing dataset included patients hospitalized between July 1, 2017 and December 31, 2017 at the same center. There was no overlap between patients in the training and testing data. Annotations per image is the average number of physicians who annotated each image in that dataset. Increase in performance between internal validation (valid) and testing may be due to more annotations resulting in an improved ARDS reference standard. AUROC: Area under the receiver operator characteristic curve; BN: Batch normalization. **The bolded CNN is the network used for all subsequent analysis**

Supplementary Table 4: CNN performance on patient subgroups in the UM test set

	N	AUROC (95% CI)	p-value	Sensitivity* (95% CI)	Specificity* (95% CI)
Overall	1560	0.92 (0.89-0.94)		77.4 (72.3-82.4)	88.7 (86.1-90.9)
Sex					
Male	899	0.91 (0.87-0.93)	0.212	73.0 (65.4-80.0)	89.8 (86.8-92.5)
Female	661	0.93 (0.90-0.96)	ref	83.3 (76.5-90.1)	87.2 (82.5-91.2)
Age					
<40	253	0.91 (0.85-0.96)	0.706	69.2 (55.9-81.6)	92.6 (87.0-96.8)
40-60	476	0.92 (0.88-0.96)	ref	80.0 (70.1-87.7)	88.5 (83.5-93.1)
60-75	652	0.91 (0.87-0.94)	0.623	77.3 (69.0-84.8)	86.6 (82.4-90.4)
>75	179	0.92 (0.85-0.97)	0.878	81.0 (62.0-93.5)	91.2 (84.9-95.6)
Race					
Black	163	0.90 (0.81-0.97)	0.629	80.0 (64.9-93.0)	86.1 (76.6-94.7)
White	1287	0.92 (0.90-0.94)	ref	77.5 (71.3-82.6)	89.5 (86.8-92.0)
Other	110	0.85 (0.67-0.97)	0.405	69.6 (40.0-90.9)	86.1 (76.6-94.7)
BMI					
<25	502	0.89 (0.83-0.93)	0.004	76.3 (66.0-84.5)	82.7 (77.5-87.4)
25-30	425	0.93 (0.89-0.96)	0.126	69.6 (40.0-90.9)	90.0 (85.2-94.1)
30-35	249	0.95 (0.92-0.98)	ref	86.1 (77.3-92.3)	93.2 (88.7-96.7)
>35	313	0.90 (0.85-0.95)	0.024	69.4 (56.6-82.1)	91.2 (86.1-95.4)

*Sensitivity and Specificity was determined using a CNN calibrated probability of 50% as the threshold for identifying chest radiographs with ARDS.

Supplementary Table 5. CNN performance in the UM test dataset based on the 3-class latent class model

	N*	AUROC (95% CI)	AUPRC (95% CI)
UM testing dataset	1253	0.96 (0.95-0.97)	0.91 (0.87-0.94)
UM testing subset reviewed by additional physicians	293	0.98 (0.96-0.99)	0.91 (0.82-0.96)

*This analysis excludes chest radiographs categorized as “uncertain” in a 3-class latent class model of ARDS, with groups: “ARDS”, “uncertain”, “Not ARDS”

Supplementary Figure 2. Calibration of the CNN in the UM internal test set

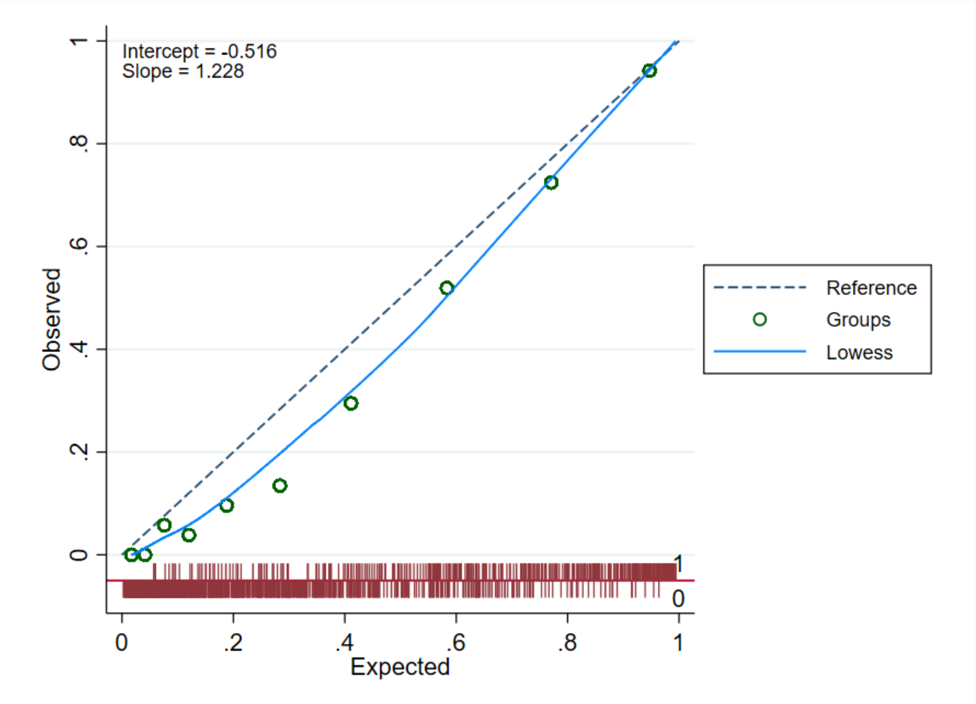

Lowess is a lowess smoothed plot of predicted probabilities and observed ARDS rates. Green circles are the mean predicted probability and observed ARDS rates for chest radiographs grouped by decile

Supplementary Figure 3. Calibration of the CNN in the UPenn external test set

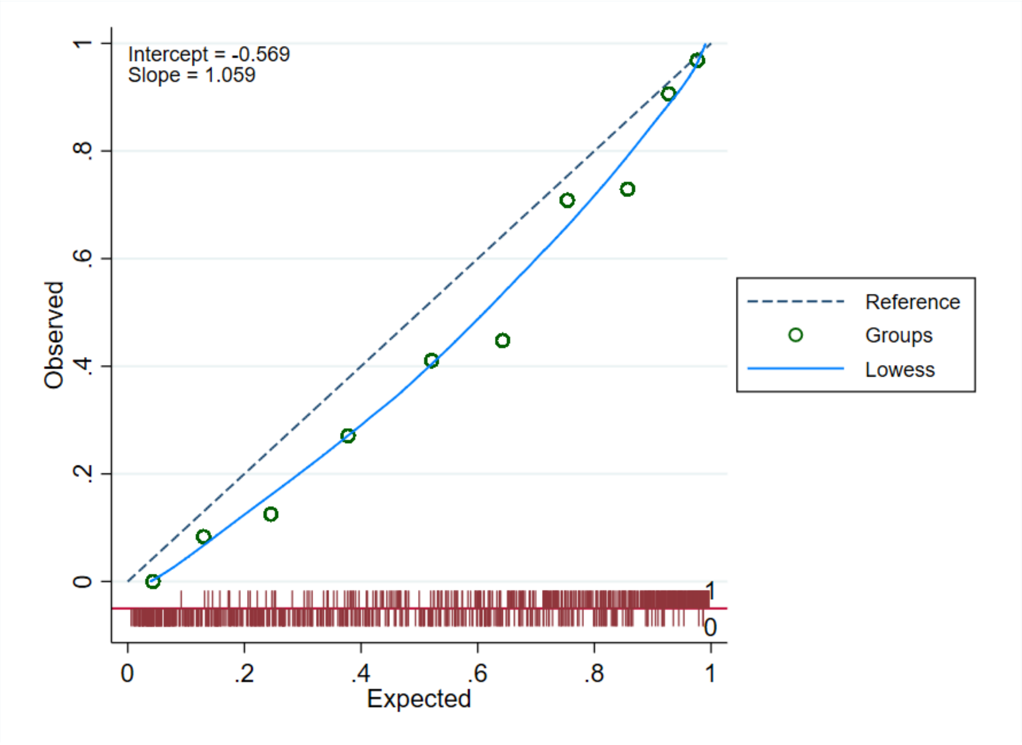

References

1. Irvin J, Rajpurkar P, Ko M, et al. CheXpert: A Large Chest Radiograph Dataset with Uncertainty Labels and Expert Comparison. *ArXiv* 2019; **abs/1901.07031**.
2. Johnson AEW, Pollard TJ, Berkowitz SJ, et al. MIMIC-CXR, a de-identified publicly available database of chest radiographs with free-text reports. *Scientific data* 2019; **6**(1): 317.
3. Sjoding MW, Hofer TP, Co I, Courey A, Cooke CR, Iwashyna TJ. Interobserver Reliability of the Berlin ARDS Definition and Strategies to Improve the Reliability of ARDS Diagnosis. *Chest* 2018; **153**(2): 361-7.
4. Cicchetti DV, Shoinralter D, Tyrer PJ. The Effect of Number of Rating Scale Categories on Levels of Interrater Reliability : A Monte Carlo Investigation. *Applied Psychological Measurement* 1985; **9**(1): 31-6.
5. Huang G, Liu Z, Weinberger KQ. Densely Connected Convolutional Networks. *2017 IEEE Conference on Computer Vision and Pattern Recognition (CVPR)* 2016: 2261-9.
6. Deng J, Dong W, Socher R, Li L, Kai L, Li F-F. ImageNet: A large-scale hierarchical image database. 2009 IEEE Conference on Computer Vision and Pattern Recognition; 2009 20-25 June 2009; 2009. p. 248-55.
7. Kingma DP, Ba J. Adam: A Method for Stochastic Optimization. *CoRR* 2015; **abs/1412.6980**.
8. Huang Y, Li W, Macheret F, Gabriel RA, Ohno-Machado L. A tutorial on calibration measurements and calibration models for clinical prediction models. *Journal of the American Medical Informatics Association : JAMIA* 2020; **27**(4): 621-33.
9. Rutter CM. Bootstrap estimation of diagnostic accuracy with patient-clustered data. *Academic radiology* 2000; **7**(6): 413-9.
